# Characterization of dental pulp stem cells isolated from a patient diagnosed with Crouzon syndrome

**DOI:** 10.1002/jcp.30241

**Published:** 2021-01-01

**Authors:** Daisuke Torii, Tomoko Kobayashi, Tetsuro Horie, Takeo W. Tsutsui

**Affiliations:** ^1^ Department of Pharmacology The Nippon Dental University School of Life Dentistry Tokyo Japan; ^2^ Research Center for Odontology The Nippon Dental University School of Life Dentistry Tokyo Japan; ^3^ Department of Developmental and Regenerative Dentistry The Nippon Dental University School of Life Dentistry Tokyo Japan; ^4^ Department of Oral Health The Nippon Dental University School of Life Dentistry Tokyo Japan

**Keywords:** Crouzon syndrome, dental pulp stem cells, fibroblast growth factor receptor 2, mitogen‐activated protein kinase, osteocalcin

## Abstract

Stem cells isolated from patients with rare diseases are important to elucidate their pathogeny and mechanisms to enable regenerative therapy. However, the mechanisms underlying tissue regeneration using patient‐derived dental pulp stem cells (DPSCs) are unclear. In this study, we investigated the levels of mRNA and protein expression related to cellular differentiation of Crouzon syndrome patient‐derived DPSCs (CS‐DPSCs) with a Gly338Arg fibroblast growth factor receptor 2 mutation. Multipotency‐related gene expression levels were equivalent in both healthy donor DPSCs and CS‐DPSCs. CS‐DPSCs showed higher *osteocalcin* (*OCN*) expression than healthy donor DPSCs. CS‐DPSCs showed a lower increase in the rate of *OCN* expression among phorbol 12‐myristate 13‐acetate (PMA)‐treated cells than healthy donor DPSCs compared with untreated control cells. CS‐DPSCs showed a lower phosphorylation rate of p38 and p44/42 in PMA‐treated cells than healthy donor DPSCs compared with untreated control cells. These results demonstrate that CS‐DPSCs have higher *OCN* expression and lower PMA stimulation‐responsiveness than healthy donor DPSCs.

## Graphical abstract

We examined the cellular response to phorbol 12‐myristate 13‐acetate stimulation to elucidate the molecular mechanisms of odontoblastic differentiation in dental pulp stem cells derived from a Crouzon syndrome patient (CS‐DPSCs) for analyzing FGF signal transduction and osteocalcin expression in CS‐DPSCs.

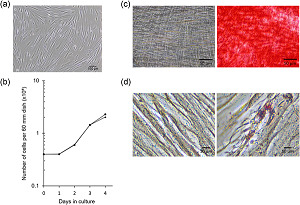

## 1 INTRODUCTION

Dental pulp stem cells (DPSCs) are somatic stem cells with a multi‐lineage differentiation potential in adult dental pulp (Gronthos et al., 2002). DPSCs have been reported to express OCT3/4, NANOG, and CD146 that are markers for cellular stemness and self‐replication (Shi et al., 2003; Fang et al., 2017; Matsui et al., 2018). In addition to their cellular stemness, as multipotency, DPSCs have been demonstrated to induce mineralization in vitro and form dentin/pulp‐like structures in vivo (Gronthos et al., 2000). Therefore, DPSCs are thought to be a promising resource for dental pulp regenerative therapy.

For the potential therapeutic applications including tissue replacement and ex vivo gene therapy using viral vectors to transduce progenitor cells, disease‐specific stem cells have many possibilities to produce cell‐processed materials for patients with rare genetic diseases (De Ravin et al., 2016; Keller et al., 2018). In the present study, we analyzed cell proliferation and differentiation of Crouzon syndrome (CS) patient‐derived DPSCs cultured in serum‐free medium to avoid the risks of xenoimmunization and zoonotic transmission for use in cellular regenerative therapy (Dessels et al., 2016).

CS is a rare craniosynostosis that presents with cranial deformity, hypoplastic maxilla, and autonomous abnormalities in osteoblast differentiation associated with fibroblast growth factor receptor (FGFR) 2 mutation in exons 3, 8, 10, 14, or 17 (Azoury et al., 2017; Kan et al., 2002). In terms of the abnormal bone formation in CS patients, some studies have reported increases in the expression level of *osteocalcin* (*OCN*) that is specifically expressed in osteoblasts and odontoblasts (Fan et al., 2018; Liu, Kwon, et al., 2013; Liu, Nam, et al., 2013).

During normal osteoblastic differentiation, fibroblast growth factor (FGF) binding to FGFR leads to FGFR dimerization and activation of mitogen‐activated protein kinases (MAPKs), p44/42, p38, and protein kinase C (PKC) that mediate the effects of FGF signaling on the phosphorylation of transcription factors and expression of downstream osteoblast differentiation markers, such as *collagen type I alpha 1* (*COL1A1*) and *OCN* (Marie, 2003).

Mutations in the FGFR2 locus have been reported to increase the expression levels and extent of phosphorylation of PKC (Fragale et al., 1999; Lomri et al., 2001) and its activity increases in osteoblasts (Lemonnier et al., 2000; Lemonnier et al., 2001). However, little is known about the relationship between FGF signaling disorders and CS pathogenesis.

Phorbol 12‐myristate 13‐acetate (PMA) induces membrane translocation and enzyme activation of PKC and the *OCN* promoter in osteoblast‐like cells (Boguslawski et al., 2000; Cheung et al., 2006).

In this study, we examined the cellular response to PMA stimulation to elucidate the molecular mechanisms of odontoblastic differentiation in DPSCs derived from a CS patient (CS‐DPSCs) to analyze FGF signal transduction and *OCN* expression in CS‐DPSCs.

## 2 METHODS

### 2.1 Cell culture

DPSCs were obtained from a patient with CS and healthy donors with approval by the Committee of Ethics, Nippon Dental University School of Life Dentistry, Tokyo. Informed consent was obtained from the patient. The dental pulp tissue was enzymatically digested as described in a previous study (Matsui et al., 2018). The cells were cultured in serum‐based minimum essential medium alpha (MEMα; Gibco/Thermo Fisher Scientific) supplemented with 20% fetal bovine serum (FBS; SAFC Biosciences; Gibco/Thermo Fisher Scientific), 100 µM *l*‐ascorbic acid phosphate magnesium salt *n*‐hydrate (Wako Pure Chemical Industries), 2 mM *l*‐glutamine (Gibco/Thermo Fisher Scientific), 100 U/ml penicillin, and 100 µg/ml streptomycin (Gibco/Thermo Fisher Scientific) at 37°C with 5% CO_2_ until Passage 1. Subsequently, the cells were cultured in STEMPRO® MSC SFM (Gibco/Thermo Fisher Scientific), a serum‐free medium for mesenchymal stem cell culture. The medium was changed every 2 days. At confluency, they were subcultured at a split ratio of 1:2 by gentle separation with TrypLE™ Express solution (Gibco/Thermo Fisher Scientific) at room temperature. To analyze the response to PMA, the cells were cultured in 60‐mm dishes (Corning), treated with 2.5 nM PMA (LC Laboratories), and harvested in centrifuge tubes. Cells cultured without PMA served as controls.

### 2.2 Cell growth assay

Cells were plated at approximately 1.91 × 10³ cells/cm² in 60‐mm dishes, and counts were presented as the mean from three dishes (*n* = 3) per time point. Cell proliferation and doubling times were determined by a logarithmic growth curve.

### 2.3 Mineralization assay

Cells were seeded at 1 × 10^4^ cells/well in 24‐well plates (Corning) and cultured until 80%–100% confluent. Then, the culture medium was changed to induction medium (MEMα supplemented with 10% FBS, 100 µM *l*‐ascorbic acid phosphate magnesium salt *n*‐hydrate, 2 mM l‐glutamine, 100 U/ml penicillin, 100 µg/ml streptomycin, 10 mM sodium β‐glycerophosphate *n*‐hydrate (Wako Pure Chemical Industries), and 10 nM dexamethasone (Wako Pure Chemical Industries)). Cellular mineralization was induced for up to 4 weeks. Cells were fixed with 4% paraformaldehyde (Wako Pure Chemical Industries) and then stained with Alizarin Red S (Merck).

### 2.4 Adipogenic differentiation assay

Cells were seeded at 5.1 × 10^4^ cells/well in six‐well plates (Corning). The culture medium was changed to adipogenic induction medium (MEMα supplemented with 20% FBS, 0.5 mM 3‐isobutyl 1‐methylxanthine (Merck), 0.5 µM hydrocortisone (Merck), 60 µM indomethacin (Merck), 100 µM *l*‐ascorbic acid, and 2 mM *l*‐glutamine). Adipogenic induction was performed for up to 2 weeks. Cells were stained with Oil Red O (Merck) at 2 weeks after adipogenic induction.

### 2.5 DNA sequencing

Genomic DNA from CS‐DPSCs was extracted using a GenElute™ Mammalian Genomic DNA Miniprep Kit (Merck), according to the manufacturer's instructions. The genomic DNA of the FGFR2 region from exon 8 to 11 was amplified by polymerase chain reaction (PCR) using a PrimeSTAR® HS DNA Polymerase Kit (Takara Bio), according to the manufacturer's instructions. Primer set (0.2 µM) was sense, 5′‐CAGCTTATTTATTGGTCTCTCATTCTC‐3′ and antisense, 5′‐CACAGAAGTCGATGGCATCAAAGCAGAG‐3′ (amplicon length: 5213 bp). The reaction was performed in an Applied Biosystems Veriti thermocycler (Thermo Fisher Scientific). The thermocycler conditions were cycled 30 times at 98°C for 10 s, 63°C for 5 s, 72°C for 5 min 12 s. Sequencing of the PCR product was performed using an Applied Biosystems 3730xl DNA Analyzer (Thermo Fisher Scientific) by Fasmac sequencing (Fasmac).

### 2.6 RNA extraction and complementary DNA synthesis

Total RNA from cells was extracted using an RNeasy® Mini Kit (QIEGAN). Complementary (cDNA) was synthesized with a High Capacity RNA‐to‐cDNA™ Kit (Thermo Fisher Scientific), according to the manufacturer's instructions.

### 2.7 Reverse transcription‐polymerase chain reaction

cDNA (0.5 µl) was diluted in a 25 µl PCR reaction mix of AmpliTaq Gold® 360 Master Mix with DNA polymerase (Thermo Fisher Scientific). Human‐specific primer sets (0.2 µM) were *OCT3/4* (sense, 5′‐CTTGCTGCAGAAGTGGGTGGAGGAA‐3′ and antisense, 5′‐CTGCAGTGTGGGTTTCGGGCA‐3′; amplicon length: 169 bp; Tan et al., 2007), *NANOG* (sense, 5′‐AGTCCCAAAGGCAAACAACCCACTTC‐3′ and antisense, 5′‐ATCTGCTGGAGGCTGAGGTATTTCTGTCTC‐3′; amplicon length: 164 bp; Tan et al., 2007), *CD146* (sense, 5′‐CCAAGGCAACCTCAGCCATGTC‐3′ and antisense, 5′‐CTCGACTCCACAGTCTGGGACGACT‐3′; amplicon length: 438 bp; Shih et al., 1998), *β‐actin* (sense, 5′‐GTCCACCTTCCAGCAGATGT‐3′ and antisense, 5′‐AAAGCCATGCCAATCTCATC‐3′, amplicon length: 165 bp; Sethi et al., 2011). The reactions were performed in the Applied Biosystems Veriti thermocycler. The thermocycler conditions for *OCT3/4* were 95°C for 9 min and then 35 cycles of 95°C for 30 s, 61°C for 30 s, and 72°C for 10 s. The conditions for *NANOG* were 95°C for 9 min and then 35 cycles of 95°C for 30 s, 62°C for 30 s, and 72°C for 10 s. The conditions for *CD146* were 95°C for 9 min and then 35 cycles of 95°C for 30 s, 60°C for 30 s, and 72°C for 26 s. The conditions for *β‐actin* were 95°C for 9 min and then 35 cycles of 95°C for 30 s, 59°C for 30 s, and 72°C for 11 s. The final extension step was 72°C for 7 min. After the PCR, 10 µl of each amplification products was analyzed by 1.5% agarose gel electrophoresis, stained with SYBR® Green I Nucleic Acid Gel Stain (Takara Bio), and visualized by a Ez‐Capture MG imaging system (ATTO).

### 2.8 Quantitative RT‐PCR

Quantoitative reverse transcription PCR (qRT‐PCR) was performed using TaqMan® Fast Advanced Master Mix (Thermo Fisher Scientific) in a StepOnePlus™ thermocycler (Thermo Fisher Scientific). The primers used for qRT‐PCR were purchased from Thermo Fisher Scientific. The primers were specific for VIC®‐conjugated *β‐actin* (4326315E; endogenous control), FAM™‐conjugated *runt‐related transcription factor 2* (*Runx2*) (assay ID: Hs00231692_m1), *OCN* (assay ID: Hs01587814_g1), transcripts of FGFR2 exons 5 and 6 (assay ID: Hs01552926_m1), and transcripts of FGFR2 exons 17 and 18 (assay ID: Hs01552921_g1, respectively; Thermo Fisher Scientific). Data were analyzed in triplicate samples by StepOne™ Software v2.3 (Thermo Fisher Scientific) and presented as relative expression of each gene compared with healthy donor DPSCs.

### 2.9 Immunoblot analysis

Cells were scraped off a 60‐mm cell culture dish and centrifuged. The cells were washed with phosphate‐buffered saline (Nissui Pharmaceutical) three times and then resuspended in radio‐immunoprecipitation assay buffer containing 1 mM phenylmethylsulfonyl fluoride, cOmplete™ protease inhibitors (Merck) and PhosSTOP™ phosphatase inhibitors (Merck) for total protein extraction. The protein contents in supernatants of the cell lysate were quantified using a Pierce™ Bicinchoninic acid Protein Assay Kit (Thermo Fisher Scientific). Then, the sample was mixed with sodium dodecyl sulfate (SDS) sample buffer and boiled for 2 min. SDS‐polyacrylamide gel electrophoresis was conducted with a 12.5% c‐PAGEL HR gel (ATTO). The proteins were transferred to Immobilon®‐P transfer membranes (Merck). After blocking the membranes with 2% ECL Prime Blocking Reagent (GE Healthcare) in tris‐buffered saline with Tween 20 (TBS‐T) at room temperature for 30 min, the proteins on the membranes were probed with primary antibodies rabbit anti‐p44/42 (sc‐94, 1/200; Santa Cruz Biotechnology TX), rabbit anti‐p38 (M0800, 1/10000; Merck), rabbit anti‐phosphorylated (p‐) p44/42 (#4370, 1/2000), anti‐p‐p38 (#4511, 1/1000; Cell Signaling Technology, Danvers, MA), or rabbit anti‐β‐actin (GTX109639, 1/5000; GeneTex at 4°C overnight. The primary antibodies were diluted with 2% ECL Prime Blocking Reagent. The membranes were washed with TBS‐T and then probed with an anti‐immunpglobulin horseradish peroxidase‐conjugated secondary antibody (1/10000; Cell Signaling Technology) at room temperature for 1 h. After three washes with TBS‐T, the labeled proteins on the membranes were detected using EzWestLumi plus chemiluminescence immunoblotting detection reagent (ATTO). The Ez‐Capture MG imaging system was applied to obtain photographs, and image analysis was performed using ImageJ (NIH, Bethesda; www.rsb.info.nih.gov/ij).

### 2.10 Statistical analysis

The statistical significance of the difference in gene expression among the groups amplified measured by qRT‐PCR was determined with the unpaired *t* test or a one‐way analysis of varince with Tukey's post hoc test, assuming independent variance.

## 3 RESULTS

### 3.1 Cell morphology, proliferation, and stem cell marker expression

**Figure 1:**
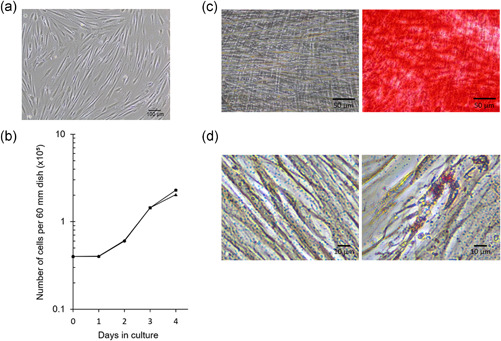
Cell morphology, proliferation, and differentiation of Crouzon syndrome patient‐derived dental pulp stem cell (CS‐DPSC)s. (a) Morphology of CS‐DPSCs. (b) Cell growth assay of CS‐DPSCs in serum‐based medium (dots) and serum‐free medium (triangles). (c) CS‐DPSCs were cultured in serum‐based medium (left panel) or mineralization induction medium (right panel) for 4 weeks and then stained with Alizarin Red S. (d) CS‐DPSCs were cultured in serum‐based medium (left panel) or adipogenic induction medium (right panel) for 2 weeks and then stained with Oil Red O

We first analyzed the cell growth curve of CS‐DPSCs cultured in serum‐based or serum‐free medium and observed cellular differentiation to evaluate cell proliferation and multipotency. Isolated CS‐DPSCs showed a fibroblast‐like morphology (Figure 1a). The proliferation rate of CS‐DPSCs in serum‐based medium was higher than that in serum‐free medium at Day 4 (Figure 1b). After 4 weeks of mineralization induction, CS‐DPSCs in serum‐based medium showed Alizarin Red S‐positive nodules (Figure 1c). CS‐DPSCs in serum‐based medium also showed Oil Red O‐positive lipid droplets after 2 weeks of adipogenic induction (Figure 1d).

**Figure 2:**
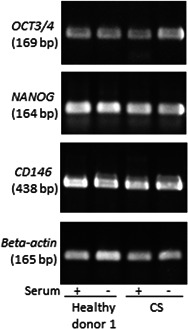
Expression of stem cell markers in healthy donor DPSCs and CS‐DPSCs. Reverse transcription‐polymerase chain reaction (RT‐PCR) assay of cells in serum‐based or serum‐free medium revealed expression of *OCT3/4*, *NANOG*, and *CD146*. *β‐Actin* expression is shown as an internal control. CS‐DPSC, Crouzon syndrome patient‐derived dental pulp stem cell

Both healthy donor DPSCs and CS‐DPSCs cultured in serum‐based and serum‐free medium showed the same extents of expression of multipotency‐related genes *OCT3/4*, *NANOG*, and *CD146* (Figure 2).

### 3.2 FGFR2 mutation and *OCN* expression

**Figure 3:**
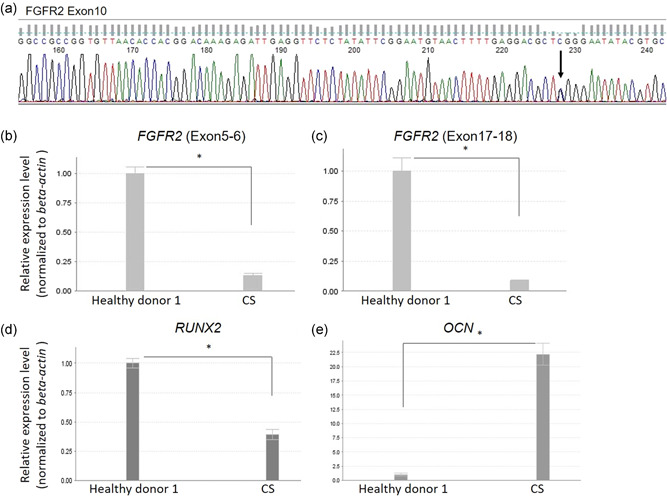
DNA sequence and quantitative RT‐PCR analyses of FGFR2. (a) Genomic DNA sequence analysis for FGFR2 exon 10 in CS‐DPSCs. Arrow indicates c.1012G>C. Quantitative RT‐PCR analysis was used to determine gene expression of (b) the coding region of FGFR2 exons 5 and 6, (c) the coding region of FGFR2 exons 17 and 18, (d) *RUNX2*, and (e) *OCN* in both healthy donor DPSCs and CS‐DPSCs. *β‐Actin* was used as an internal control. Statistical significance was determined by the unpaired *t* test (*p < .05). CS‐DPSC, Crouzon syndrome patient‐derived dental pulp stem cell; RT‐PCR, reverse transcription‐polymerase chain reaction

Next, we performed genomic DNA sequencing of the FGFR2 region in CS‐DPSCs amplified by PCR, because CS has been reported to be caused by FGFR2 mutation (Kan et al., 2002; Zhang et al., 1999). The DNA sequencing identified a heterozygous missense mutation c.1012G>C, p. Gly338Arg (G338R) in FGFR2 exon 10 (Figure 3a). FGFR2 exons 8–18 had no mutation except for exon 10 (data not shown).

Next, we examined the gene expression levels of the coding region of the extracellular and intracellular domains of FGFR2 to evaluate positional effects of the point mutation (Kleinjan & van Heyningen, 1998). The gene expression level of the coding region of FGFR2 exons 5 and 6, a part of the extracellular domains of the receptor, was 0.14‐fold lower in CS‐DPSCs than in healthy donor DPSCs (Figure 3b). The gene expression level of the coding region of exons 17 and 18, a part of the intracellular domains of the receptor, was 0.09‐fold lower in CS‐DPSCs (Figure 3c).

The gene expression level of *Runx2*, a transcription factor regulating osteogenesis, was 0.4‐fold lower in CS‐DPSCs than in healthy donor DPSCs (Figure 3d). Conversely, *OCN* expression was 22.0‐fold higher in CS‐DPSCs than in healthy donor DPSCs (Figure 3e).

### 3.3 *OCN* upregulation by PMA treatment

**Figure 4:**
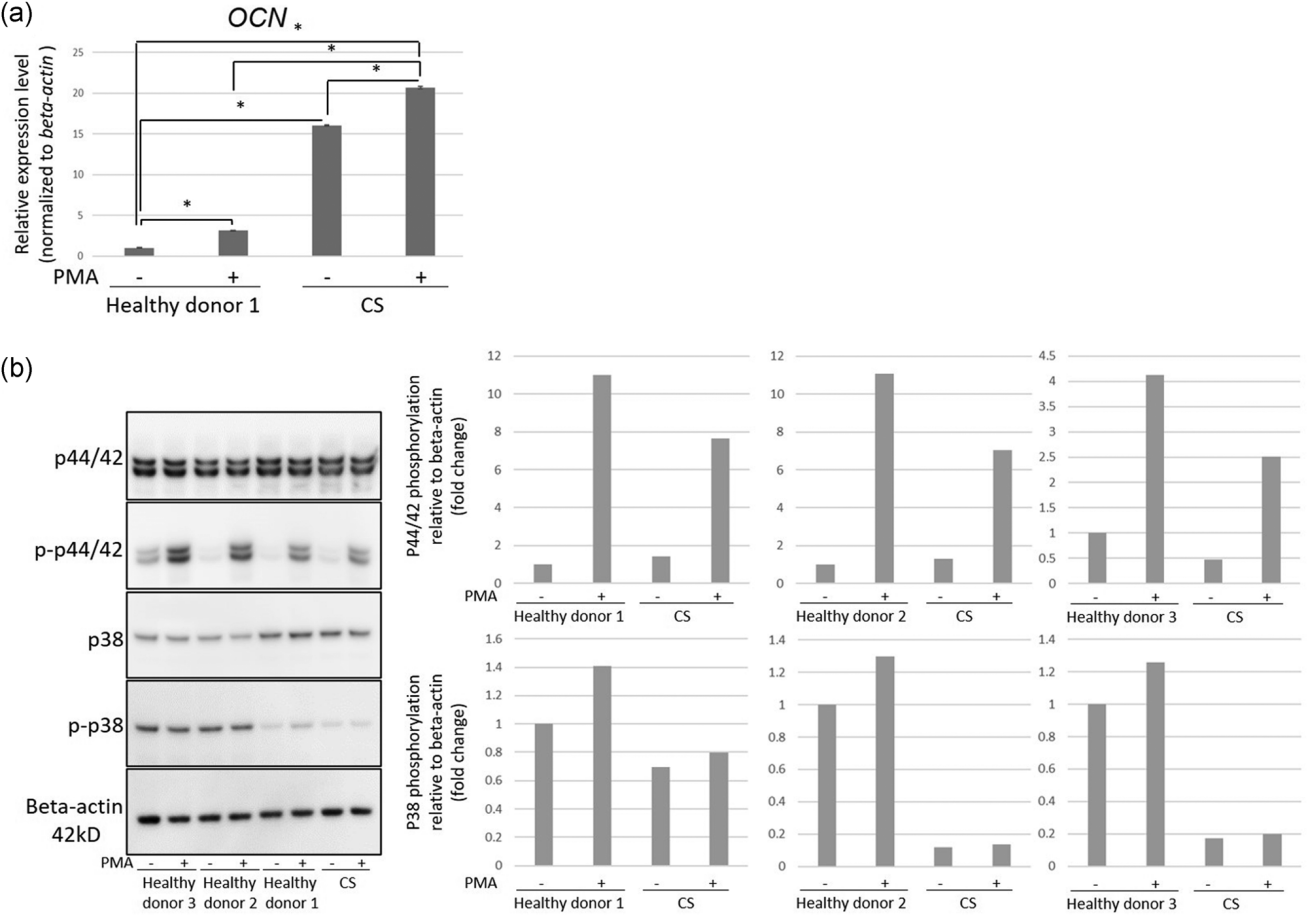
Effects of PMA on DPSCs. (a) *OCN* expression in healthy donor DPSCs and CS‐DPSCs after 8 h of stimulation with or without PMA. *β‐Actin* was used as an internal control. The graph shows the mean ± *SD* of triplicates. Statistical analyses were performed by a one‐way ANOVA with Tukey's post hoc test (*p < .0001). (b) Immunoblot analysis for p44/42, p‐p44/42, p38, and p‐p38 in healthy donor DPSCs and CS‐DPSCs after 30 min of stimulation with or without PMA. The graph shows the ratio of the phosphorylated protein to the total protein on blot images normalized to β‐actin. ANOVA, analysis of variance; CS‐DPSC, Crouzon syndrome patient‐derived dental pulp stem cell; PMA, phorbol 12‐myristate 13‐acetate

We next assessed *OCN* expression in healthy donor DPSCs and CS‐DPSCs treated with PMA to detect cellular responses downstream of FGF signaling. In healthy donor DPSCs after 8 h of stimulation with PMA, *OCN* expression was 3.15‐fold higher in PMA‐treated cells than in cells without PMA treatment, whereas *OCN* expression in PMA‐treated CS‐DPSCs was 1.29‐fold higher in CS‐DPSCs without PMA treatment (Figure 4a). After 4 h of stimulation with PMA, *OCN* expression was 1.19‐fold higher in healthy donor DPSCs treated with PMA than in healthy donor DPSCs without PMA treatment, whereas *OCN* expression in PMA‐treated CS‐DPSCs did not increase (data not shown). After 1 h of stimulation with PMA, *OCN* expression in healthy donor DPSCs and CS‐DPSCs did not increase (data not shown).

We finally examined phosphorylation of p44/42 and p38 in healthy donor DPSCs and CS‐DPSCs after 30 min of stimulation with PMA to detect rapid protein kinase reactions. The phosphorylation ratio of p44/42 in CS‐DPSCs was 0.14–0.43‐fold lower than in healthy donor DPSCs after PMA stimulus (Figure 4b). Moreover, the phosphorylation ratio of p38 in CS‐DPSCs was 0.14–0.63‐fold lower than in healthy donor DPSCs after PMA stimulus (Figure 4b).

## 4 DISCUSSION

DPSCs are present in adult dental pulp and obtained from teeth extracted for clinical reasons. They have a rapid proliferation potency and cellular stemness (Shi et al., 2005). Hence, they are expected to be useful for dental pulp regenerative therapy, and it is important to characterize patient‐specific stem cells. Here, we investigated the characteristics of CS‐DPSCs in vitro for possible regenerative therapy of patients.

CS is a craniosynostosis that presents with limb anomalies and craniofacial dysmorphologies. Treatment is typically surgical correction and prevention of future deformations. Further research efforts are needed for effective methods of early intervention and prevention (Azoury et al., 2017).

In the present study, we found that CS‐DPSCs showed cell proliferation, differentiation (Figure 1a–d), and multipotency gene expression in vitro (Figure 2). CS‐DPSCs also formed dentin/pulp‐like structures by engraftment into immunocompromised mice (data not shown). Recent studies have reported a high proliferation rate and multipotency in DPSCs (Fang et al., 2017; Matsui et al., 2018). Accordingly, CS‐DPSCs are thought to have a high proliferation potency and cellular stemness, and they expressed a high level of *OCN* mRNA (Figure 3e).

CS is caused by FGFR2 mutation (Kan et al., 2002; Zhang et al., 1999), but the details of the pathological mechanisms remain unclear. Understanding the molecular mechanisms has allowed for investigation of various therapeutic agents that can potentially be used to prevent the disorder (Azoury et al., 2017). Therefore, it is necessary to analyze FGF signal transduction and the molecular profile in CS‐DPSCs. We found that the FGFR2 mutation point of CS‐DPSCs was c.1012G>C, p. G338R, in exon 10, which encodes the amino terminal portion of the extracellular immunoglobulin‐like III domain (Fan et al., 2018; Gorry et al., 1995), by DNA sequence analysis (Figure 3a). G338R FGFR2 mutation was recently reported to cause high expression of osteogenic markers, such as *OCN* in the orbital bone (Fan et al., 2018). Our data also demonstrated high expression of *OCN* in CS‐DPSCs compared with healthy donor DPSCs (Figure 3e). Conversely, gene expression of the coding region of the extracellular and intracellular domains of FGFR2 was lower in CS‐DPSCs than in healthy donor DPSCs (Figure 3b,c). Thus, the high expression of *OCN* in CS‐DPSCs was thought to occur because of the G338R FGFR2 mutation, while the point mutation decreased the transcription of other exons in FGFR2. The cause for the gene expression profile requires further investigation.

*OCN* expression is regulated via PKC and MAPK pathways downstream of FGFR signaling (Marie, 2003). Several studies have reported that PMA activates PKC and induces *OCN* expression in osteoblast‐like cells (Boguslawski et al., 2000; Cheung et al., 2006). In calvarial osteoblasts, the S252W FGFR2 Apert mutation increases expression of osteoblastic differentiation marker genes via highly activated PKC, p38, and p44/42 (Lemonnier et al., 2001; Suzuki et al., 2012). The Cys342Tyr FGFR2 CS mutant is also reported to activate p44/42 (Lee et al., 2018; Pfaff et al., 2016), but abnormal osteoblastic gene expression via PKC and MAPK signaling in G338R FGFR2 CS mutants were still unknown.

Our results showed a low increase rate of *OCN* expression and low phosphorylation rate of p38 and p44/42 in PMA‐treated CS‐DPSCs compared with healthy donors DPSCs (Figure 4a,b). These findings suggest that G338R FGFR2 CS mutants have dysfunction in repression of *OCN* expression in the resting status and show low PMA stimulation responsiveness downstream of FGFR signaling compared with normal cells.

In terms of clinical implications, we assessed the characteristics of CS‐DPSCs for possible cellular regenerative therapies of maxillofacial and dental malformations. For treatment of skeletal dysplasia, previous studies have shown that the soluble form of mutant FGFR2 and adeno‐associated virus‐mediated RNA interference partially prevent craniosynostosis in the Apert syndrome mouse model (Luo et al., 2018; Morita et al., 2014), while allogeneic mesenchymal stem cells have been used for engraftment in patients with osteogenesis imperfecta (Le Blanc et al., 2005). We demonstrated that CS‐DPSCs possessed a mineralization ability in the present study.

This study provides new insights into the molecular mechanism of CS pathogenesis and the possibility of regenerative therapy using cells from patients.

## AUTHOR CONTRIBUTIONS

Daisuke Torii, and Takeo W. Tsutsui designed the study. Daisuke Torii, Tomoko Kobayashi, Tetsuro Horie, and Takeo W. Tsutsui performed the experiments and analyzed the data. All authors read and approved the manuscript.

## CONFLICT OF INTERESTS

The authors declare that there are no conflict of interests.
